# Molecular taxonomy of the two *Leishmania* vectors *Lutzomyia umbratilis* and *Lutzomyia anduzei* (Diptera: Psychodidae) from the Brazilian Amazon

**DOI:** 10.1186/1756-3305-6-258

**Published:** 2013-09-11

**Authors:** Vera Margarete Scarpassa, Ronildo Baiatone Alencar

**Affiliations:** 1 Laboratório de Genética de Populações e Evolução de Mosquitos Vetores, Coordenação de Biodiversidade, Instituto Nacional de Pesquisas da Amazônia, Avenida André Araujo, 2.936, Bairro Petrópolis, Manaus CEP 69.067-375, Amazonas, Brazil; 2 Laboratório de Flebotomíneos e Triatomíneos, Coordenação de Biodiversidade, Instituto Nacional de Pesquisas da Amazônia, Avenida André Araujo, 2.936. Bairro Petrópolis, Manaus CEP 69.067-375, Amazonas, Brazil

**Keywords:** *Leishmania* vectors, Barcode region, Molecular entomology, Speciation

## Abstract

**Background:**

*Lutzomyia umbratilis* (a probable species complex) is the main vector of *Leishmania guyanensis* in the northern region of Brazil. *Lutzomyia anduzei* has been implicated as a secondary vector of this parasite. These species are closely related and exhibit high morphological similarity in the adult stage; therefore, they have been wrongly identified, both in the past and in the present. This shows the need for employing integrated taxonomy.

**Methods:**

With the aim of gathering information on the molecular taxonomy and evolutionary relationships of these two vectors**,** 118 sequences of 663 base pairs (barcode region of the mitochondrial DNA cytochrome oxidase I – *COI*) were generated from 72 *L. umbratilis* and 46 *L. anduzei* individuals captured, respectively, in six and five localities of the Brazilian Amazon. The efficiency of the barcode region to differentiate the *L. umbratilis* lineages I and II was also evaluated*.* The data were analyzed using the pairwise genetic distances matrix and the Neighbor-Joining (NJ) tree, both based on the Kimura Two Parameter (K2P) evolutionary model.

**Results:**

The analyses resulted in 67 haplotypes: 32 for *L. umbratilis* and 35 for *L. anduzei*. The mean intra-specific genetic distance was 0.008 (0.002 to 0.010 for *L. umbratilis*; 0.008 to 0.014 for *L. anduzei*), whereas the mean interspecific genetic distance was 0.044 (0.041 to 0.046), supporting the barcoding gap. Between the *L. umbratilis* lineages I and II, it was 0.009 to 0.010. The NJ tree analysis strongly supported monophyletic clades for both *L. umbratilis* and *L. anduzei*, whereas the *L. umbratilis* lineages I and II formed two poorly supported monophyletic subclades.

**Conclusions:**

The barcode region clearly separated the two species and may therefore constitute a valuable tool in the identification of the sand fly vectors of *Leishmania* in endemic leishmaniasis areas. However, the barcode region had not enough power to separate the two lineages of *L. umbratilis*, likely reflecting incipient species that have not yet reached the status of distinct species.

## Background

Phlebotomine sand flies (Diptera: Psychodidae) are vectors of human leishmaniasis, a disease caused by trypanosomatids of the genus *Leishmania*. *Leishmania* infection is characterized by a species-specific pathology, varying from cutaneous lesions to the potentially fatal visceral form [1,2]. This disease occurs in the tropical, subtropical and Mediterranean regions of the world and its global burden has been estimated to be ~500,000 cases of visceral leishmaniasis (VL) and ~1.1-1.5 million cases of cutaneous leishmaniasis (CL) per year [2,3]. Out of the six genera belonging to the subfamily Phlebotominae, only *Lutzomyia* and *Phlebotomus* include the vectors of human leishmaniasis. The former is restricted to the New World, where approximately 32 out of more than 500 species described [4] are implicated as vectors, whereas the latter is found in the Old World [1,5]. In the New World, *Lutzomyia* (*Lutzomyia*) *longipalpis* (likely a species complex) is recognized as the main vector of visceral leishmaniasis in the Neotropics [6,7], whereas *Lutzomyia* (*Nyssomyia*) *umbratilis*, *Lutzomyia* (*Nyssomyia*) *flaviscutellata, Lutzomyia* (*Nyssomyia*) *whitmani* sensu lato, *Lutzomyia* (*Nyssomyia*) *intermedia* sensu lato, *Lutzomyia* (*Nyssomyia*) *neivai*, *Lutzomyia* (*Nyssomyia*) *olmeca*, *Lutzomyia* (*Lutzomyia*) *gomezi, Lutzomyia* (*Psychodopygus*) *wellcomei*, *Lutzomyia* (*Viannamyia) furcata*, among others, are important vectors of cutaneous leishmaniasis [7].

*Lutzomyia umbratilis* is a highly anthropophilic sand fly that has been appointed as the main vector of *Leishmania guyanensis*, the etiological agent of cutaneous leishmaniasis (CL) in northern Brazil [8-14] and probably in other countries of northern South America [15-18]. *Lutzomyia anduzei*, its probable sister taxon, is also an anthropophilic species [9,19]. Arias and Freitas [11] isolated *Leishmania* spp. (likely *Le. guyanensis*) from wild-caught *L. anduzei* females collected near the city of Manaus, in the State of Amazonas, Brazil. Similar findings were also reported in the State of Pará, Brazil [9]. Hence, the sporadic records of infections by *L. anduzei* suggest that it could be a secondary vector in the Brazilian Amazon [7,9,19]*.* There are actually only a few studies on *L. anduzei*, and the available data are limited to species diversity and abundance in areas of leishmaniasis transmission. Consequently, only little is known about the accurate geographic distribution, ecology and genetics of this species and its efficiency as vector of this parasite. Additionally, the data published regarding *L. anduzei* before *L. umbratilis* had been described may in fact refer to the latter [8,20].

*Lutzomyia anduzei* and *L. umbratilis* are two closely related species exhibiting high morphological similarity in the adult stage [19,21]. Both species are geographically distributed in northern South America, with extensive overlapping areas [19]. *Lutzomyia anduzei* was described by Rozeboom [22], who used specimens (females) from Gran Sabana in Venezuela. *Lutzomyia umbratilis* was described by Ward and Fraiha [23], based on specimens captured in the Jari River region, State of Pará, Brazil. Because of the high morphological similarity between them, *L. umbratilis* has been wrongly identified as *L. anduzei* in the past [8,20]. This misidentification may still occur today, compromising the accurate identification of the vectors involved. Along with the difficulty of separating these species, recent studies have demonstrated that *L. umbratilis* may represent a cryptic species complex of at least two distinct or incipient species which are separated across opposite banks of the largest rivers in the central region of the Brazilian Amazon [24,25] and are probably different in vector competence [11]. Therefore, the use of molecular markers combined with morphology (integrated taxonomy) could make the identification of *L. umbratilis* and *L. anduzei* more accurate and differentiate the two lineages within *L. umbratilis*, which in turn is relevant for understanding the epidemiology and the distinct patterns of transmission of *Le. guyanensis,* thus facilitating the vector control efforts in this region.

### Main morphological and chromatic differences between *L. umbratilis* and *L. anduzei*

**Figure 1:**
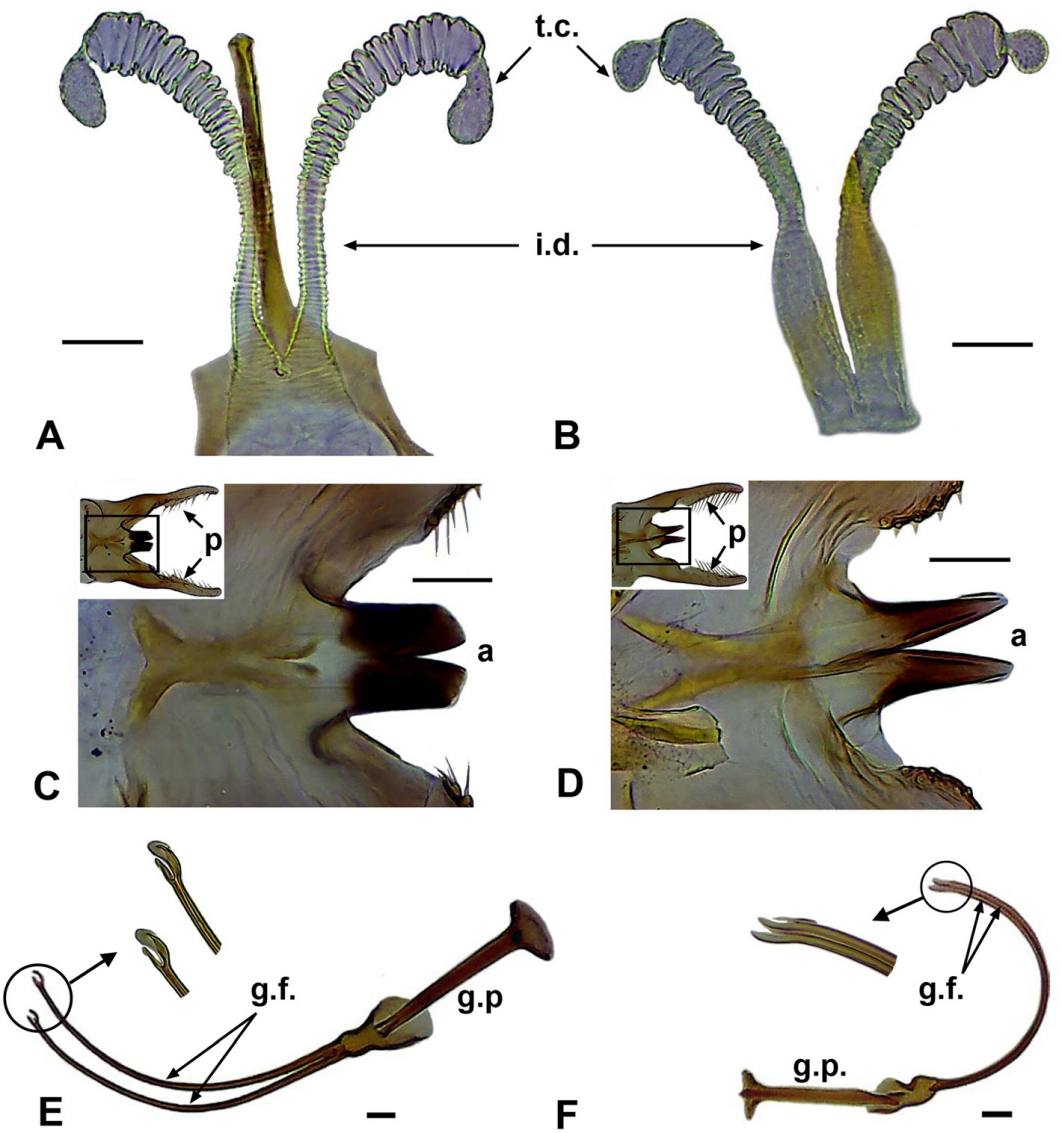
**Female and male internal and external genitals of *Lutzomyia umbratilis *and *Lutzomyia anduzei*. A** and **B**: Spermathecae of *L. umbratilis* and *L. anduzei* females*,* respectively; **C** and **D**: Aedeagi of *L. umbratilis* and *L. anduzei* males, respectively (upper left corners, showing the position of aedeagi regarding the parameres); **E** and **F**: Genital filaments apex of *L. umbratilis* and *L. anduzei* males*,* respectively. t.t.: terminal tubercles; i.d.: individual ducts; p: parameres; a: aedeagus; g.f.: genital filaments; g.p.: genital pumps. The arrows indicate in highlight the apex of the genital filaments. Bar = 20 μm.

The morphological differences between *L. umbratilis* and *L. anduzei* are subtle [19, R. A. Freitas, personal communication] (Figure 1). The adults of these species can show slight differences in color, with *L. umbratilis* exhibiting a light brown body and *L. anduzei* an almost pale coloration. The females of *L. umbratilis* have a well developed spermatheca compared to those of *L. anduzei*. In the latter, the terminal tubercle of this organ (spermatheca head) is thinner than the terminal ring. In *L. umbratilis*, the common and individual ducts show sharper transverse striations (Figure 1A), whereas in *L. anduzei* these striations are weak and fade toward the common duct (Figure 1B). Furthermore, in *L. anduzei* there is an evident narrowing of individual ducts at the junction with the body. As for the males, in *L. umbratilis*, the aedeagus apex is truncated (Figure 1C), whereas in *L. anduzei* it is slender (Figure 1D). The apex of the genital filament is slightly bifid in *L. umbratilis* males (Figure 1E) but bezel-shaped in *L. anduzei* males (Figure 1F).

The barcode region comprising 648 base pairs (bp) at the 5′ end of the mitochondrial DNA (mtDNA) cytochrome oxidase I - *COI* has emerged as the standard region for the identification of a wide variety of invertebrate and vertebrate species [26], although failures have been reported due to non-monophyly [27]. This region has shown to be exceptionally promising for species-level identification in insects [28-30], including *Anopheles*[31,32] and other mosquitoes [33]. However, the barcode region has so far been little investigated in sand flies in general [34] and not at all in sand flies from Brazil.

In the Brazilian Amazon region, although there are areas of high transmission of leishmaniasis [35] and of sand fly vectors presenting morphological variations as well as isomorphic taxa [7], studies on molecular taxonomy, phylogenetic relationships, molecular evolution and population genetics are scarce [5,25,36,37], and nothing is known about the genetic diversity of these sand flies, which could contribute to their management. Hence, the aim of this study was to seek information on the molecular taxonomy and to preliminarily clarify the evolutionary relationships of *L. umbratilis* and *L. anduzei* using the DNA barcode region to help in their accurate identification and, thereby better determine the role of each one in the leishmaniasis foci. Additionally, this study assessed the efficiency of this fragment in differentiating the two lineages or incipient *L. umbratilis* species, previously described by Scarpassa and Alencar [25].

## Methods

### Samples and collection sites

**Figure 2:**
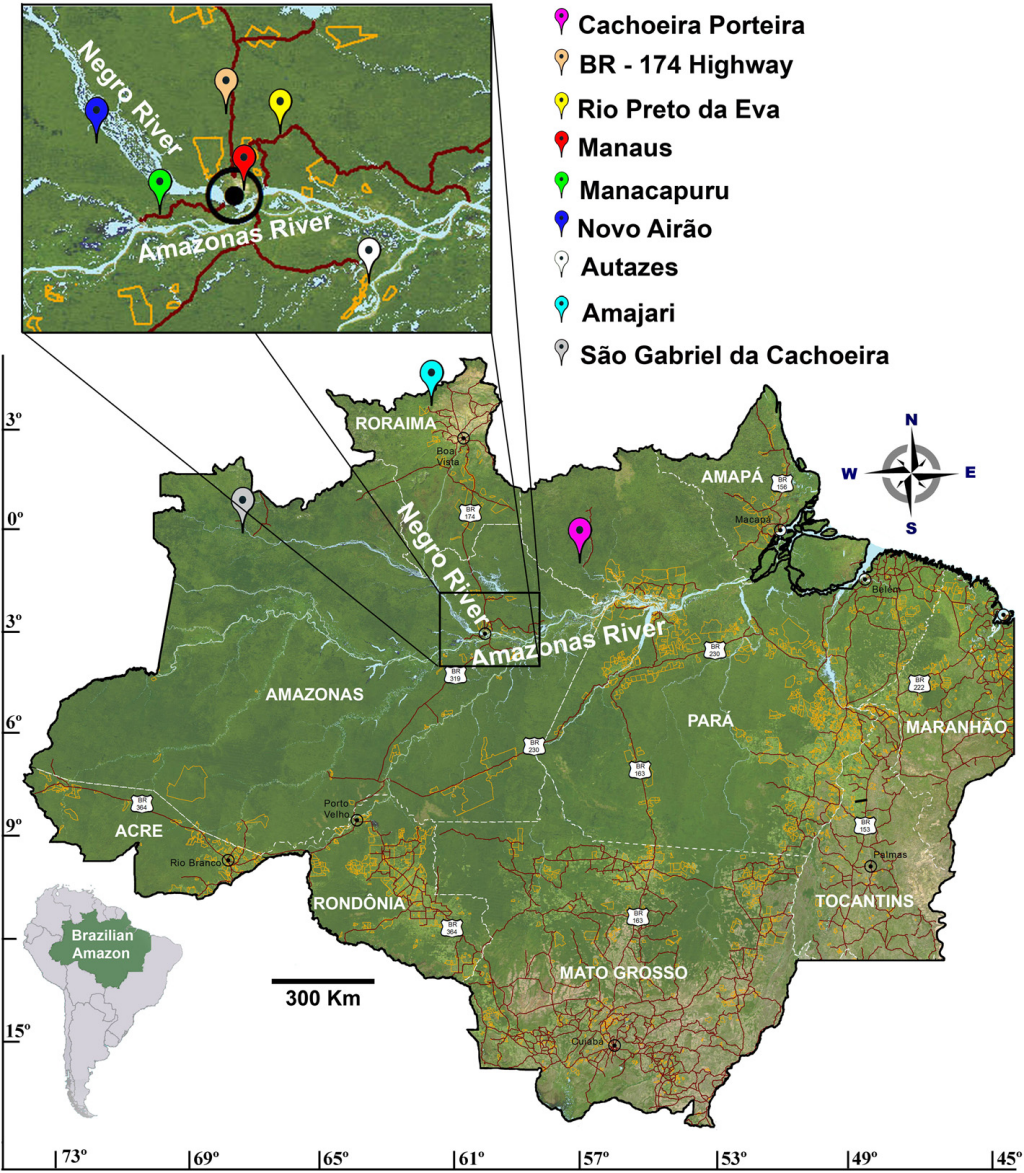
**Collection sites of *Lutzomyia umbratilis *and *Lutzomyia anduzei *samples from the Brazilian Amazon.***Lutzomyia umbratilis*: Cachoeira Porteira, BR-174 Highway, Rio Preto da Eva, Manaus, Manacapuru and Novo Airão. *Lutzomyia anduzei*: Manaus, Autazes, Novo Airão, Amajari and São Gabriel da Cachoeira.

*Lumbratilis umbratilis* adults were collected in six localities of the Brazilian Amazon region (Table 1, Figure 2), including Cachoeira Porteira in the State of Pará; km 43 of the BR-174 Highway; km 65 of the AM-010 Highway in the municipality of Rio Preto da Eva; a fragment of urban forest in Manaus; km 60 of the AM-070 Highway in the municipality of Manacapuru; and km 60 and km 70 of the AM-352 Highway in the municipality of Novo Airão, in the State of Amazonas, as described in Scarpassa and Alencar [25]. The collection sites of Cachoeira Porteira, BR-174 Highway, Rio Preto da Eva and Manaus are situated on the left bank of the Negro River and north of the Amazonas River, and the samples from these sites were named lineage I. The localities of Manacapuru and Novo Airão are situated on the right bank of the Negro River and the samples from these sites were named lineage II [25]. *Lutzomyia anduzei* adults were captured in five locations of the Brazilian Amazon, comprising four sites in the State of Amazonas (urban forest fragment in Manaus; municipality of Autazes; km 60 and km 70 of the AM-352 Highway in the municipality of Novo Airão; municipality of São Gabriel da Cachoeira), and one in the State of Roraima (Amajari) (Table 1, Figure 2). The two species were captured sympatrically in Manaus and Novo Airão. All information regarding collection data, coordinates and sample size of the species is displayed in Table 1.

**Table 1:** **Localities data and haplotype frequency of the *Lutzomyia umbratilis *and *Lutzomyia anduzei *samples from the Brazilian Amazon**

**Species**	**Localities, State**	**Co-ordinates (Lat., Long.)**	**N**	**Haplotype frequency**
*Lutzomyia umbratilis*	Cachoeira Porteira, Oriximiná, Pará	1° 28′ S; 56° 22′ W	12	H1(1), H2(2), H3(1), H4(1), H5(1), H6(1), H7(1), H8(1), H9(1), H10(2)
	Km 43 of BR-174 Highway, Amazonas	2° 36′ S; 60° 02′ W	12	H11(1), H12(1), H13(6), H14(1), H15(1), H16(1), H17(1)
	Rio Preto da Eva, Amazonas	2° 43′ S; 59° 47′ W	13	H12(5), H13(5), 18(1), H19(1), H20(1)
	Manaus, Amazonas	3° 04′ S; 59° 57′ W	12	H13(3), H15(1), H19(4), H21(1), H22(1), H23(1), H24(1)
	Manacapuru, Amazonas	3° 14′ S; 60° 31′ W	13	H25(2), H26(8), H27(1), H28(1), H29(1)
	Novo Airão, Amazonas	2° 47′ S; 60° 55′ W	10	H26(5), H29(2), H30(1), H31(1), H32(1)
**Subtotal**			**72**	
*Lutzomyia anduzei*	Manaus, Amazonas	3° 04′ S; 59° 57′ W	10	H33(1), H34(2), H35(1), H36(1), H37(1), H38(1), H39(1), H40(1), H41(1)
	Autazes, Amazonas	3º 42′ S; 59º 07′ W	2	H42(1), H43(1)
	Novo Airão, Amazonas	2° 47′ S; 60° 55′ W	13	H42(2), H44(6), H45(2), H46(1), H47(1), H48(1)
	Amajari, Roraima	3º 46′ N; 61º 44′ W	8	H49(1), H50(1), H51(2), H52(1), H53(1), H54(1), H55(1)
	São Gabriel da Cachoeira, Amazonas	0º 03′ S; 66º 59′ W	13	H41(1), H56(1), H57(1), H58(1), H59(1), H60(1), H61(1), H62(1), H63(1), H64(1), H65(1), H66(1), H67(1)
**Subtotal**			**46**	
**TOTAL**			**118**	

*N* sample size, *H1 to H32* Haplotypes observed in the samples of *Lutzomyia umbratilis*, *H33 to H67* Haplotypes observed in the samples of *Lutzomyia anduzei*. Inside the parentheses is the number of individuals observed for each haplotype. The underlined haplotypes are shared among localities.

A 663 base pairs (bp) fragment (DNA barcode) was generated from 72 *L. umbratilis* and 46 *L. anduzei* specimens captured in the Brazilian Amazon region (Table 1). Adults of both species were collected with CDC (Centers for Disease Control) miniature light traps and with aspirators placed on the bases of tree trunks, as described in Scarpassa and Alencar [25]. This study and catch protocol was reviewed and approved by the Institutional Review Board of the National Institute of Amazonian Research (INPA), of the Brazilian Ministry of Science, Technology and Innovation (MCTI). The sample collections were authorized by the Brazilian Institute for the Environment and Renewable Natural Resources (IBAMA) and by the System of Authorization and Information in Biodiversity (SISBIO), license number 12733–1 for the collection of *L. umbratilis* and *L. anduzei* from Amazonas and Roraima States, Brazil, and license number 14054–5 for the collection of *L. umbratilis* from the Cachoeira Porteira, State of Pará, Brazil. After collection, the sand flies were preserved in 95% ethanol and stored at −20°C until processing for DNA extraction. Morphological identification of the species was based on the internal and external genitalia of males and females [19], using an optical microscope at a magnification of 10x, 40x and 100x (Carl Zeiss, Primo Star, 3119000947, Germany). The male and female genitalia of *L. umbratilis* and *L. anduzei* shown in Figure 1 are from specimens collected in Cachoeira Porteira (State of Pará) and Autazes (State of Amazonas), respectively (see Table 1).

### DNA extraction, PCR and sequencing

Total genomic DNA was extracted individually from whole sand flies using the phenol and chloroform method [38], resuspended in 20 μL of 1x TE buffer (10 mM Tris-Cl pH 8.0, 1 mM EDTA pH 8.0) or sterile water, and then stored at −80°C until amplification by polymerase chain reaction (PCR). The primers (10 μM) used in the amplification reaction were LCO 1490 and HCO 2198 [39]. All PCR reactions included negative controls. The PCR products were visualized on 1% agarose gels under UV light, purified with PEG and both DNA strands were sequenced in an ABI 3130 XL Automated DNA Sequencer (Applied Biosystems), available at INPA (Manaus, Brazil).

### Data analysis

All sequences were automatically aligned with the Clustal W and then compiled and edited in BIOEDIT v. 7.0.8.0 [40]. Following, sequence identity searches were performed using BLAST (Basic Local Alignment Search Tool), available at http://www.ncbi.nlm.nih.gov/BLAST/. The haplotype numbers for each species, number of polymorphic sites and other measures of genetic diversity were estimated using the DNASP v. 5.10 [41] and TCS v. 1.21 [42] softwares. The nucleotide frequencies, numbers of transitions and transversions, the number of variable sites among haplotypes, and intra and interspecific genetic distances (sequence divergence), based on the Kimura Two Parameter (K2P) evolutionary model, were calculated in MEGA v. 5.1 [43]. The phylogenetic relationship of the haplotypes was inferred using a Neighbor Joining (NJ) tree, with 2,000 replicates, and constructed based on the K2P distances. The tree was rooted using sequences of *Anopheles triannulatus* sensu lato (Diptera: Culicidae: Anophelinae) (V. M. Scarpassa, unpublished data) as outgroup.

The 663 bp barcode region analyzed in this study overlapped between sites 283 and 663 with those of *L. umbratilis* analyzed by Scarpassa and Alencar [25] who studied a 1181 bp fragment of the *COI* gene. Haplotypes sequences are deposited in GenBank under the accession numbers KF467531 to KF467597.

## Results

The DNA barcode region analyzed in this study comprised 663 bp. All alignments were unambiguous and no insertions or deletions were detected in the dataset. The amino acid translations showed no stop codons, ensuring that the dataset did not constitute nuclear mitochondrial DNA sequences (NUMTs). The amino acid reading frame starts at the first base of the primer-edited sequences. All sequences, including those from *L. umbratilis* and *L. anduzei* (n=118), yielded 75 (11.31%) variable sites and 53 (8%) of these were parsimoniously informative. Transitions were more common than transversions (Table 2). The average nucleotide composition was similar in the two species, with an overall average of 38.2% for Thymine (T), 30.3% for Adenine (A), 15.4% for Cytosine (C) and 16.2% for Guanine (G). The A+T content were rich (68.5%), as observed in other insects.

**Table 2:** **Mean of nucleotide frequencies, and transitions and transversions rate of the *Lutzomyia umbratilis*, *Lutzomyia anduzei *and total**

	**Identical pairs**	**TS (%)**	**TV (%)**	**T**	**C**	**A**	**G**	**Total bases**
***L. umbratilis***								
Average	659	4	0	38.2	15.2	30.5	16.1	663
1st	221	0	0	28	14.9	28	29.4	221
2nd	221	0	0	43	26.2	13.6	17.6	221
3rd	217	4	0	44	4.6	50	1.1	221
***L. anduzei***								
Average	656	6	1	38.2	15.6	29.9	16.3	663
1st	220	1	0	28	14.5	28	29.4	221
2nd	221	0	0	43	26.2	13.6	17.6	221
3rd	215	5	1	44	6	48.2	1.8	221
**Total general**								
Average	647	12	4	38.2	15.4	30.3	16.2	663
1st	220	1	0	28	14.8	28	29.4	221
2nd	221	0	0	43	26.2	13.6	17.6	221
3rd	206	11	4	44	5.1	49.3	1.4	221

*TS* number of transitions, *TV* number of transversions.

One hundred and eighteen sequences resulted in a total of 67 haplotypes (Table 1), reflecting a high haplotype diversity for the two species. Out of 32 haplotypes (H1-H32) observed for 72 *L. umbratilis* specimens, six (H12, H13, H15, H19, H26, H29) were common to the analyzed localities. The sample of Cachoeira Porteira, situated at the north of the Amazonas River (lineage I), however, did not share any haplotype with other localities, likely due to the geographic distance. The samples from the three other locations (BR-174 Highway, Rio Preto da Eva and Manaus), situated on the left bank of the Negro River (also lineage I), shared the haplotypes H12, H13, H15 and H19, suggesting gene flow. The samples from Manacapuru and Novo Airão, situated on the right bank of the Negro River (lineage II), shared the haplotypes H26 and H29. H13 was the most frequent in lineage I, whereas the H26 was the most frequent in lineage II. No haplotypes were shared between samples from opposite river banks (lineages I and II), as previously detected by Scarpassa and Alencar [25].

The analysis of 46 *L. anduzei* specimens yielded 35 haplotypes (H33-H67) (Table 1). Of these, only two (H41 and H42) were shared among samples. The samples from Manaus and São Gabriel da Cachoeira, geographically distant, shared H41, whereas the samples from Autazes and Novo Airão shared H42. The sample from Amajari did not share any haplotype with another locality. Additional file 1 shows the nucleotide substitutions among 67 haplotypes, where ten fixed mutations (four transitions, at positions 366, 369, 538 and 660; six transversions, at positions 45, 102, 222, 459, 528 and 558) between *L. umbratilis* and *L. anduzei* were observed. *Lutzomyia umbratilis* lineages I and II, however, had one fixed mutation (a T↔C transition at position 21) and three almost fixed mutations (all A↔G transitions, at positions 540, 567 and 624). Table 3 portrays the summary of genetic diversity measures for *L. umbratilis* and *L. anduzei*, which were higher in the latter.

**Table 3:** **Summary of genetic diversity measures of *Lutzomyia umbratilis *and *Lutzomyia anduzei***

**Summary statistics**	***L. umbratilis***	***L. anduzei***
Nº of sequences (*N*)	72	46
Nº of polymorphic sites (*S*)	28	48
Total Nº of mutations	29	51
Nº of haplotypes	32	35
Haplotype diversity (*h*) ± SD	0.921 ± 0.019	0.979 ± 0.012
Nucleotide diversity (*π*) ± SD	0.00602 ± 0.00025	0.01070 ± 0.00096
Average nucleotide differences (*k*)	3.993	7.095

*SD* Standard deviation.

Table 4 shows the pairwise intra and interspecific genetic distances (K2P) of the two species. The average intraspecific genetic distance was 0.008, ranging from 0.002 to 0.010 in *L. umbratilis* and from 0.008 to 0.014 in *L. anduzei*. The mean interspecific genetic distance between *L. umbratilis* and *L. anduzei* was ~six-fold higher (0.044 ± 0.007; varying from 0.041 to 0.046), supporting the barcoding gap. Within species *L. umbratilis*, the genetic distance between lineages I and II varied from 0.009 to 0.010, corroborating the previous observations of Scarpassa and Alencar [25].

**Table 4:** **Mean intra and interspecific genetic distances, based on the K2P, of the *Lutzomyia umbratilis *and *Lutzomyia anduzei *samples**

**Samples**	**N**	**CPumbr**	**BRumbr**	**RPumbr**	**MNumbr**	**MCumbr**	**NAumbr**	**MNandu**	**AUandu**	**NAandu**	**AJandu**	**SGandu**
CPumbr	12	**0.005**										
BRumbr	12	0.005	**0.003**									
RPumbr	13	0.005	0.002	**0.002**								
MNumbr	12	0.005	0.004	0.004	**0.003**							
MCumbr	13	0.009	0.010	0.009	0.009	**0.002**						
NAumbr	10	0.009	0.010	0.009	0.009	0.002	**0.001**					
MNandu	10	0.042	0.043	0.043	0.043	0.044	0.044	**0.007**				
AUandu	2	0.041	0.041	0.041	0.042	0.042	0.042	0.008	**0.011**			
NAandu	13	0.045	0.045	0.045	0.045	0.046	0.046	0.012	0.011	**0.014**		
AJandu	8	0.043	0.044	0.044	0.044	0.044	0.044	0.009	0.010	0.014	**0.010**	
SGandu	13	0.042	0.044	0.043	0.043	0.044	0.044	0.008	0.011	0.014	0.010	**0.007**

*CP* Cachoeira Porteira, *BR* BR-174 Highway, *RP* Rio Preto da Eva, *MN* Manaus, *MC* Manacapuru, *NA* Novo Airão, *AU* Autazes, *AJ* Amajari, *SG* São Gabriel da Cachoeira, *umbrLutzomyia umbratilis*, *anduLutzomyia anduzei*, *N* sample sizes. Values in **bold** indicate genetic distances between haplotypes intra-sample. The underlined values are related to comparisons between samples of the lineages I and II of *Lutzomyia umbratilis*.

**Figure 3:**
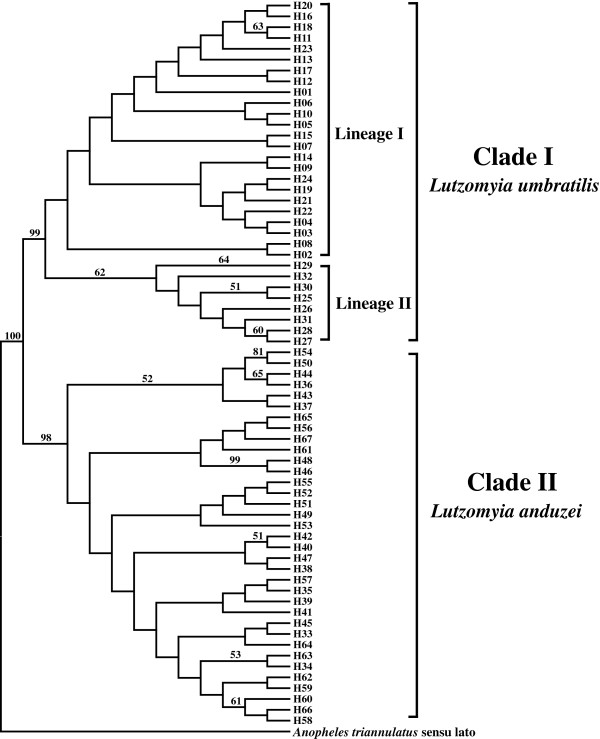
**Neighbor Joining (NJ) tree of the 67 haplotypes observed in *Lutzomyia umbratilis *and *Lutzomyia anduzei*, inferred under the Kimura 2 Parameter (K2P) model.** Numbers above branch represent bootstrap supports. Values lower than 50 are not represented on branch. Sequences of *Anopheles triannulatus* sensu lato were used as outgroup.

The phylogenetic relationships of haplotypes visualized in the NJ tree (Figure 3), using K2P, supported monophyly for *L. umbratilis* and *L. anduzei*, with bootstrap values of 99% and 98%, respectively, indicating that these species can be recognized by their barcode region. Clade I, comprising *L. umbratilis* lineages I and II, formed two monophyletic subclades, but had low bootstrap support. Lineage I haplotypes (H1 to H24) were clustered in several subdivisions, whereas all haplotypes of lineage II (H25 to H32) were grouped in another subclade, with bootstrap support of 62%. Clade II, comprising *L. anduzei* haplotypes (H33 to H67), consisted of smaller subdivisions, regardless of the geographic origin of the haplotypes (Table 1), but was also poorly supported.

## Discussion

The phylogenetic relationships of haplotypes suggest that *L. umbratilis* and *L. anduzei* are monophyletic, with strongly supported clades; therefore, the barcode region can be used for differentiating these two species. The genetic distance between them was rather small (4.4%) and similar to the values found between members of species complexes in mosquitoes [31,32,44]. Barcode region analysis provided resolution for 13 sand fly species of genus *Lutzomyia*, with the probable existence of cryptic species in two of them [34]; the authors, however, did not mention the genetic distances among the species studied. The genetic distance observed in this study is supported by the great morphological similarity between *L. umbratilis* and *L. anduzei*[19]. Scarpassa and Alencar [25] observed a higher value (5.8%) for the genetic distance between *L. umbratilis* and *L. anduzei*. These differences may be explained by the fact that the *COI* fragment at the 3′ end analyzed by these authors was more variable, resulting in higher differentiation. Additionally, in this study the most of the transitions and transversions observed between *L. umbratilis* and *L. anduzei* occurred in the third codon position, except for one transition, located in the first codon position (Table 2). Taken together, these findings may suggest that *L. umbratilis* and *L. anduzei* are either closely related species or of a recent common origin. However, our data brought no evidence of genetic introgression between them, at least at the mitochondrial DNA level.

The phylogenetic relationships observed in this study for the *L. umbratilis* lineages I and II indicated two monophyletic subclades, but were poorly supported statistically. The fragment of end 3′ analyzed by Scarpassa and Alencar [25] generated trees with better resolution and two moderately supported monophyletic clades, likely due to its higher variation level. On the other hand, biological and morphological differentiation [24,45] and probable differences in vector competence [11] have been described for lineages I and II. In contrast, preliminary data obtained with nuclear markers are indicating few differences between the two lineages (V. M. Scarpassa and R. B. Alencar, unpublished data), which could be the result of recent speciation, where the differences are visible only in mitochondrial genes.

In the present study, the value of the genetic distance between *L. umbratilis* lineages I and II was low (0.9-1.0%), identical to the one found previously by Scarpassa and Alencar [25]. This distance falls within the range of intraspecific nucleotide differences, which has been less than 2% among Diptera species for the barcode region [31], but there is an exception [46]. However, the absence of shared haplotypes detected in the present and in previous studies [25] may indicate lack of gene flow between lineages and may be an indicator of genetic discontinuity. Species that have diverged very recently are expected to share ancestral variation in high proportions, a situation that may confound their phylogenetic reconstruction; hence, the fixed differences between these species may be evidenced only in genes involved in the speciation process [47,48]. Taken together, the low genetic distance and the poorly supported monophyletic clades observed in the present study, combined with the previous results, may suggest that two lineages of *L. umbratilis* are incipient species [49]. Whether or not these lineages consist of cryptic species within *L. umbratilis* requires further inquiry. Moreover, this differentiation between two *L. umbratilis* lineages, although low, could probably affect the genes that control vector competence and consequently lead to distinct patterns of *Le. guyanensis* transmission in the central region of the Brazilian Amazon, a hypothesis initially raised by Arias and Freitas [11]. Altogether, these data allows us to propose that the populations belonging to lineage I (left bank of the Negro River and north of the Amazonas River) could be susceptible to the development (i. e., could be vectors) of *Le. guyanensis*, whereas the populations belonging to lineage II (right bank of the Negro River and south of the Amazonas River) are likely not to be involved in the transmission (not vectors) or could be less susceptible to the development of *Le. guyanensis*. However, further studies, including entomological parameters and transmission dynamics (anthropophily and natural infection rate of samples from the field) and experimental infections of these populations are needed to either support or refute this hypothesis.

The findings of this study (Tables 1 and 3) indicated that *L. anduzei* has a greater genetic diversity than its closely related species, *L. umbratilis*, at least with regard to the barcode region. Out of the 35 haplotypes observed in *L. anduzei*, only two were shared among the samples analyzed, suggesting restricted gene flow between them. Curiously, the genetic distance observed in *L. anduzei* were slightly larger than those detected between lineages I and II of *L. umbratilis*, probably indicating that the *L. anduzei* populations consist of a significant genetic structure or display a large effective population size (*N*e) [50] or highly divergent haplotypes [51]. In fact, highly divergent haplotypes were detected in the sample from Novo Airão, consequently the comparisons involving this sample yielded the highest genetic distance (0.012, 0.014) and intra-sample (0.014) values (Table 4).

Similar to what was found for *L. umbratilis* in the present and a previous study [25], no haplotype was shared among the *L. anduzei* samples from opposite banks of the Negro and Amazonas Rivers. This raises the hypothesis that the large Rivers of the Amazon region may act as possible barriers to the sand fly species, as already discussed by Scarpassa and Alencar [25]. All these findings, including the high haplotype diversity with a great number of unique haplotypes and the strong evidence of genetic structure, demonstrate the need of population genetics studies in *L. anduzei* across its range.

## Conclusions

This is the first study of molecular taxonomy in two *Leishmania* vectors, *L. umbratilis* and *L. anduzei*, from the Brazilian Amazon region. We consider the *COI* barcode region to be a robust marker for differentiating sand fly species, even those closely related, and it may constitute a valuable tool for epidemiologic studies and for leishmaniasis control programs throughout South America. The barcode region, however, had not enough power to separate the two lineages of *L. umbratilis*, which may indicate that they are incipient species that have not yet reached the status of distinct species [49].

## Competing interests

The authors declare that they have no competing interests.

## Authors’ contributions

VMS: Designed the experiment, generated the results, analyzed the data, and wrote the manuscript. RBA: Captured the sand flies in the field, identified the samples, generated and processed morphological analyses and revised the manuscript. All authors read and approved the final version of the manuscript.

## Supplementary Material

Additional file 1 **Variable sites of the 67 haplotypes observed for *Lutzomyia umbratilis *and *Lutzomyia anduzei***. Click here for file

## References

[B1] Lane RP Lane RP, Crosskey RW Sandflies Medical insects and arachnids 1993 London: Chapman and Hall 78 119

[B2] WHO First WHO report on neglected tropical diseases: working to overcome the global impact of neglected tropical diseases 2010 Geneva: World Health Organization 172 Available at: http://www.who.int/neglected_diseases

[B3] Reithnger R Leishmaniases’ burden of disease: ways forward for getting from speculation to reality PLoS Negl Trop Dis 2008 2 e285 doi:10.1371/journal.pntd.0000285 10.1371/journal.pntd.0000285 18958280 PMC2570250

[B4] Shimabukuro PHF Galati EAB Checklist dos Phlebotominae (Diptera, Psychodidae) do Estado de São Paulo, Brasil, com comentários sobre sua distribuição geográfica Biota Neotrop 2011 11 http://www.biotaneotropica.org.br/v11n1a/pt/abstract?inventory +bn0361101a2011

[B5] Mazzoni CJ Gomes CA Souza NA Queiroz RG Justiniano SCB Ward RD Kyriacou CP Peixoto AA Molecular evolution of the *period* gene in sandflies J Mol Evol 2002 55 553 562 10.1007/s00239-002-2351-z 12399929

[B6] Lainson R Ward RD Shaw JJ Experimental transmission of *Leishmania chagasi* causative agent of neotropical visceral leishmaniasis by the sandfly *Lutzomyia longipalpis* Nature 1977 266 628 630 10.1038/266628a0 859627

[B7] Lainson R Espécies neotropicais de *Leishmania*: uma breve revisão histórica sobre sua descoberta, ecologia e taxonomia Rev Pan-Amaz Saude 2010 1 2 13 32

[B8] Lainson R Ward D Shaw JJ Cutaneous leishmaniasis in North Brazil: *Lutzomyia anduzei* as a major vector Trans R Soc Trop Med Hyg 1976 70 171 172 10.1016/0035-9203(76)90202-9 960211

[B9] Lainon R Shaw JJ Ward RD Ready PD Naiff RD Leishmaniasis in Brazil: XIII. Isolation of *Leishmania* from armadillos (*Dasypus novemcinctus*), and observation on the epidemiology of cutaneous leishmaniasis in north Pará State Trans R Soc Trop Med Hyg 1979 73 239 242 10.1016/0035-9203(79)90225-6 473314

[B10] Lainson R Shaw JJ Ward RD Ready PD Miles M Póvoa MM Leishmaniasis in Brazil: XVI. Isolation and identification of *Leishmania* species from sandflies, wild mammals and man in north Pará State, with particular reference to *L. braziliensis guyanensis* causative agent of “pian-bois” Trans R Soc Trop Med Hyg 1981 75 530 536 10.1016/0035-9203(81)90192-9 7324128

[B11] Arias JR Freitas RA Sobre os vetores da leishmaniose cutânea na Amazônia central do Brasil. 2. Incidência de flagelados em flebotomíneos selváticos Acta Amaz 1978 8 387 396

[B12] Pinheiro FG Luz SLB Franco AMR Infecção natural por tripanosomatídeos (Kinetoplastida: Trypanosomatidae) em *Lutzomyia umbratilis* (Diptera: Psychodidae) em áreas de leishmaniose tegumentar americana no Amazonas, Brasil Acta Amaz 2008 38 165 172 10.1590/S0044-59672008000100019

[B13] Azevedo ACR Costa SM Pinto MCG Souza JL Cruz HC Vidal J Rangel EF Studies on the sand fly fauna (Diptera: Psychodidae: Phlebotominae) from transmission areas of American Cutaneous Leishmaniasis in state of Acre, Brazil Mem Inst Oswaldo Cruz 2008 103 760 767 10.1590/S0074-02762008000800003 19148413

[B14] Gil LHS Araújo MS Villalobos JM Camargo LMA Ozaki LS Fontes CJF Ribolla PEM Katsuragawa TH Cruz RM Silva AA Silva LHP Species structure of sand fly (Diptera: Psychodidae) fauna in the Brazilian western Amazon Mem Inst Oswaldo Cruz 2009 104 955 959 10.1590/S0074-02762009000700002 20027459

[B15] Le Pont F Pajot FX La leishmaniose em Guyane Française. 1. Étude de l´écologie et du taux d’infection naturelle de *Lutzomyia* (*Nyssomyia*) *umbratilis* Ward et Fraiha, 1977 em saison seche. Considérations épidémiologiques Cahiers ORSTOM Série Ent Méd et Parasitologie 1980 18 359 382

[B16] Gentile B Le Pont F Pajot FX Besnard R Dermal leishmaniasis in French Guiana: the sloth (*Choloepus didactylus*) as reservoir host Trans R Soc Trop Med Hyg 1981 75 612 613 10.1016/0035-9203(81)90223-6 7324144

[B17] Pajot FX Le Pont F Gentile B Besnard R Epidemiology of leishmaniasis in French Guiana Trans R Soc Trop Med Hyg 1982 76 112 113 10.1016/0035-9203(82)90033-5 7080142

[B18] Feliciangeli MD Pérez JR Ramirez A First Venezuelan record of *Lutzomyia umbratilis* Ward & Fraiha 1977 (Diptera: Psychodidae), a proven vector of *Leishmania braziliensis guyanensis* Trans R Soc Trop Med Hyg 1985 79 878 3832500 10.1016/0035-9203(85)90150-6

[B19] Young DG Duncan NA Guide to the identification and geographic distribution of *Lutzomyia* sandflyies in Mexico, the West Indies, Central and South America (Diptera: Psychodidade) Mem Am Entomol Inst 1994 54 1 881

[B20] Almeida FB Flebótomos da Amazônia. I – Sobre a presença de *Lutzomyia anduzei* (Rozeboom, 1942) no Brasil (Diptera: Psychodidade) Boletim do Instituto Nacional de Pesquisas da Amazônia: Série Patologia Tropical 1970 3 1 16

[B21] Azevedo ACR Lainson R Souza AA Fé NF Feliciangeli DM Menezes CRV Rangel EF Comparative studies of populations of *Lutzomyia umbratilis* (Diptera: Psychodidae) in Brazil and Venezuela J Med Entomol 2002 39 587 600 10.1603/0022-2585-39.4.587 12144289

[B22] Rozeboom LE *Phlebotomus anduzei*, a new phlebotomus from Venezuela Bol Ent Venez 1942 1 91 94 **In:** Floch H, Abonnenc E: **Phlébotomos de La Guyane Française. – (X) Sur lês famelles a 5° segment dês palpes court. Description du male de *****P. anduzei***. *Inst Pasteur Guyane Territ Inini Publ* 1944, **88**: 22 pp

[B23] Ward RD Fraiha H *Lutzomyia umbratilis*, a new species of sandfly from Brazil (Diptera: Psychodidae) J Med Entomol 1977 14 313 317

[B24] Justiniano SCB Chagas AC Pessoa FAC Queiroz RG Comparative biology of two populations of *Lutzomyia umbratilis* (Diptera: Psychodidae) of central Amazonia, Brazil, under laboratory conditions Braz J Biol 2004 64 227 235 10.1590/S1519-69842004000200007 15462295

[B25] Scarpassa VM Alencar RB *Lutzomyia umbratilis*, the Main Vector of *Leishmania guyanensis*, Represents a Novel Species Complex? PLoS ONE 2012 7 5 e37341 doi:10.1371/journal.pone.0037341 10.1371/journal.pone.0037341 22662146 PMC3356248

[B26] Hebert PDN de Waard JR Biological identification through DNA barcode Proc R Soc Lond B 2003 270 313 322 10.1098/rspb.2002.2218

[B27] Cognato AI Standard percent DNA sequence difference for insects does not predict species boundaries J Econ Entomol 2006 99 1037 1045 10.1603/0022-0493-99.4.1037 16937653

[B28] Hajibabaei M Janzen DH Burns JM Hallwachs W Hebert PD DNA barcodes distinguish species of tropical Lepidoptera Proc Natl Acad Sci USA 2006 103 968 971 10.1073/pnas.0510466103 16418261 PMC1327734

[B29] Smith MA Wood DM Jansen DH Hallwachs W Hebert PDN DNA barcodes affirm that 16 species of apparently generalist tropical parasitoid flies (Diptera: Tachnidae) are not all generalists Proc Natl Acad Sci USA 2007 104 4967 4972 10.1073/pnas.0700050104 17360352 PMC1821123

[B30] Virgilio M Backeljau T Nevado B De Meyer M Comparative performances of DNA barcoding across insets orders BMC Bioinforma 2010 11 206 10.1186/1471-2105-11-206 PMC2885370 20420717

[B31] McKeon SN Lehr MA Wilkerson RC Ruiz JF Sallum MA Lima JB Póvoa MM Conn JE Lineage divergence detected in the malaria vector *Anopheles marajoara* (Diptera: Culicidae) in Amazonian Brazil Malar J 2010 9 271 10.1186/1475-2875-9-271 20929572 PMC2959070

[B32] Ruiz-Lopes F Wilkerson RC Conn JE Mckeon SN Levin DM Quiñones ML Póvoa MM Linton YM DNA barcoding reveals both known and novel taxa in the *Albitarsis* group (*Anopheles*: *Nyssorhynchus*) of Neotropical malaria vectors Parasit Vectors 2012 5 44 10.1186/1756-3305-5-44 22353437 PMC3350407

[B33] Cywinska A Hunter FF Hebert PD Identifying Canadian mosquito species through DNA barcodes Med Vet Entomol 2006 20 413 424 10.1111/j.1365-2915.2006.00653.x 17199753

[B34] Azpurua J de la Cruz D Valderama A Windsor D *Lutzomyia* sandfly diversity and rates of infection by *Wolbachia* and an exotic *Leishmania* species on Barro Colorado Island, Panama PLoS Negl Trop Dis 2010 4 3 e627 doi:10.1371/journal.pntd.0000627 10.1371/journal.pntd.0000627 20231892 PMC2834748

[B35] Guerra JAO Talhari S Paes MG Garrido M Talhari JM Aspectos clínicos e diagnósticos da leishmaniose tegumentar americana em militares simultaneamente expostos à infecção na Amazônia Rev Soc Bras Med Trop 2003 36 587 590 10.1590/S0037-86822003000500008 14576873

[B36] Ready PD Day JC de Souza AA Rangel EF Davies CR Mitochondrial DNA characterization of populations of *Lutzomyia whitmani* (Diptera: Psychodidae) incriminated in the peri-domestic and silvatic transmission of *Leishmania* species in Brazil Bull Entom Res 1997 87 187 195 10.1017/S0007485300027346

[B37] Lins RMMA Oliveira SG Souza NA Queiroz RG Justiniano SCB Ward RD Kyriacou CP Peixoto AA Molecular evolution of the *cacophony* IVS6 region in sandflies Insect Mol Biol 2002 11 2 117 122 10.1046/j.1365-2583.2002.00315.x 11966876

[B38] Sambrook J Russell DW A Laboratory Manual 2001 New York: Cold Spring Harbor Laboratory Press

[B39] Folmer O Black M Hoeh W Lutz R Vrijenhoek R DNA primers for amplification of mitochondrial Cytochrome C Oxidase subunit I from diverse metazoan invertebrates Mol Mar Biol Biotechnol 1994 3 294 299 7881515

[B40] Hall TA BioEdit: a user-friendly biological sequence alignment editor and analysis program for Windows 95/98/NT Nucl Acids Symp Ser 1999 41 95 98

[B41] Librado P Rozas J DnaSP v. 5: A software for comprehensive analysis of DNA polymorphism data Bioinformatics 2009 25 1451 1452 10.1093/bioinformatics/btp187 19346325

[B42] Clement M Posada D Crandall KA TCS: a computer program to estimate gene genealogies Mol Ecol 2000 9 1657 1660 10.1046/j.1365-294x.2000.01020.x 11050560

[B43] Tamura K Peterson D Peterson N Stecher G Nei M Kumar S Mega5: Molecular evolutionary genetics analysis using maximum likelihood, evolutionary distance, and maximum parsimony methods Mol Biol Evol 2011 28 10 2731 2739 10.1093/molbev/msr121 21546353 PMC3203626

[B44] Scarpassa VM Conn JE Molecular differentiation in natural populations of *Anopheles oswaldoi* sensu lato (Diptera: Culicidae) from the Brazilian Amazon, using sequences of the *COI* gene from mitochondrial DNA Genetics Mol Res 2006 5 493 502 17117365

[B45] Justiniano SCB Biologia comparada de populações de Lutzomyia umbratilis (Diptera: Psychodidae) da Amazônia Central Brasileira 2004 Manaus, Amazonas, Brazil: Doctoral Thesis. Instituto Nacional de Pesquisas da Amazônia 151

[B46] Moreno M Bickersmith S Harlow W Hildebrandt J McKeon SN Silva-do- Nascimento TF Loaiza JR Ruiz F Lourenço-de-Oliveira R Sallum MAM Bergo ES Fritz GN Wilkerson RC Linton YM Juri MJD Rangel Y Póvoa MM Gutiérrez-Builes LA Correa MM Conn JE Phylogeography of the neotropical *Anopheles triannulatus* complex (Diptera: Culicidae) supports deep structure and complex patterns Parasit Vectors 2013 6 47 10.1186/1756-3305-6-47 23433428 PMC3606328

[B47] Krzywinski J Besansky NJ Molecular Systematic of *Anopheles*: From Subgenera to Subpopulations Annu Rev Entomol 2003 48 111 139 10.1146/annurev.ento.48.091801.112647 12208816

[B48] Moreno M Marinotti O Krzywinski J Tadei WP James AA Achee NL Conn JE Complete mtDNA genomes of *Anopheles darlingi* and an approach to anopheline divergence time Malaria J 2010 9 127 10.1186/1475-2875-9-127 PMC2877063 20470395

[B49] Queiroz K Species concepts and species delimitation Syst Biol 2007 56 879 886 10.1080/10635150701701083 18027281

[B50] Velzen RV Weitschek E Felici G Bakker FT DNA barcoding fo recently diverged species: relative performance of matching methods PLoS ONE 2012 7 1 e30490 doi:10.1371/journal.pone.0030490 10.1371/journal.pone.0030490 22272356 PMC3260286

[B51] Scarpassa VM Conn JE Mitochondrial DNA detects a complex evolutionary history with Pleistocene Epoch divergence for the Neotropical Malaria Vector *Anopheles nuneztovari* Sensu Lato Am J Trop Med Hyg 2011 85 857 867 10.4269/ajtmh.2011.11-0150 22049039 PMC3205631

