# Supplementary material for: Molecular taxonomy of the two Leishmania vectors Lutzomyia umbratilis and Lutzomyia anduzei (Diptera: Psychodidae) from the Brazilian Amazon
Source: Parasit Vectors. 2013 Sep 11;6:258. doi: 10.1186/1756-3305-6-258 (PMC3847350; doi:10.1186/1756-3305-6-258)
Supplement: Additional file 1 — Variable sites of the 67 haplotypes observed for Lutzomyia umbratilis and Lutzomyia anduzei. [file 1756-3305-6-258-S1.doc]

**Additional file 1 Variable sites of the 67 haplotypes observed for *Lutzomyia umbratilis* and *Lutzomyia anduzei***
